# LINC00853 contributes to tumor stemness of gastric cancer through FOXP3-mediated transcription of PDZK1IP1

**DOI:** 10.1186/s12575-023-00213-2

**Published:** 2023-07-04

**Authors:** Xia Hu, Maoyuan Zhao, Shuangyuan Hu, Qingsong Liu, Wenhao Liao, Lina Wan, Feng Wei, Fangting Su, Yu Guo, Jinhao Zeng

**Affiliations:** 1 TCM Regulating Metabolic Diseases Key Laboratory of Sichuan Province, Hospital of Chengdu University of Traditional Chinese Medicine, Chengdu, 610072 China; 2 School of Chinese Materia Medica, Beijing University of Chinese Medicine, 100029 Beijing, China; 3 Department of Gastroenterology, Hospital of Chengdu University of Traditional Chinese Medicine, Chengdu, 610072 China; 4 Department of Oncology, Hospital of Chengdu University of Traditional Chinese Medicine, Chengdu, 610072 China

**Keywords:** LINC00853, FOXP3, PDZK1IP1, Tumor stemness, Gastric cancer

## Abstract

**Background:**

The incidence and mortality of gastric cancer (GC) are high worldwide. Tumor stemness is a major contributor to tumorigenesis and development of GC, in which long non-coding RNAs (lncRNAs) are deeply involved. The purpose of this study was to investigate the influences and mechanisms of LINC00853 in the progression and stemness of GC.

**Methods:**

The level of LINC00853 was assessed based on The Cancer Genome Atlas (TCGA) database and GC cell lines by RT-PCR and in situ hybridization. An evaluation of biological functions of LINC00853 including cell proliferation, migration, and tumor stemness was conducted via gain-and loss-of-function experiments. Furthermore, RNA pull-down and RNA immunoprecipitation (RIP) assay were utilized to validate the connection between LINC00853 and the transcription factor Forkhead Box P3 (FOXP3). Nude mouse xenograft model was used to identify the impacts of LINC00853 on tumor development.

**Results:**

We identified the up-regulated levels of lncRNA-LINC00853 in GC, and its overexpression correlates with poor prognosis in GC patients. Further study indicated that LINC00853 promoted cell proliferation, migration and cancer stemness while suppressed cell apoptosis. Mechanistically, LINC00853 directly bind to FOXP3 and promoted FOXP3-mediated transcription of PDZK1 interacting protein 1(PDZK1IP1). Alterations of FOXP3 or PDZK1IP1 reversed the LINC00853-induced biological effects on cell proliferation, migration and stemness. Moreover, xenograft tumor assay was used to investigate the function of LINC00853 in vivo.

**Conclusions:**

Taken together, these findings revealed the tumor-promoting activity of LINC00853 in GC, expanding our understanding of lncRNAs regulation on GC pathogenesis.

**Supplementary Information:**

The online version contains supplementary material available at 10.1186/s12575-023-00213-2.

## Introduction

There are more than one million new cases of gastric cancer (GC) every year worldwide, making it one of the most common types of cancer [1, 2]. Clinical prognosis for advanced gastric cancer patients remain unsatisfactory despite advances in diagnosis and treatment [3, 4].Accumulating evidences have demonstrated that cancer cells could show stem cell -like characteristics of self-renewal ability and differentiation potential, which are recognized as cancer stem-like cells (CSCs) [5, 6]. CSCs have been identified to be involved in the poor outcomes with enhancing recurrence and drug resistance, etc. [7–9]. However, there is still a lack of understanding of the molecular mechanisms of GC tumorigenesis, metastasis, and tumor stemness. Hence, diminishing CSCs is expected to improve the efficacy of GC treatment.

LncRNAs, as a class of noncoding RNAs, feature over 200 nucleotides in length and no capacity to code for proteins [10]. LncRNA levels could be altered in human diseases including cancers [11, 12], and regulation of the expression of lncRNAs has been proposed as a promising therapeutic target for cancers [13]. Lnc-PDZD7 was identified as a negative regulator of liver cancer and contributed to the prognosis and TACE response [14]. Lnc-PDZD7 regulated cancer stemness and drug resistance through EZH2 to promoting DNA methylation of ATOH8 [14]. Through its regulation of glycolysis and cancer stemness, the lncRNA FGF13-AS1 disrupts c-Myc-IGF2BPs protein interactions in breast cancer cells [15]. Tumor-derived exosomal Lnc-Sox2ot accelerated epithelial-mesenchymal transition (EMT) and stemness through competitively binding to miR-200, which regulated the downstream target gene of SOX2 [16]. In spite of extensive research on dysregulated lncRNAs in GC, there is still no complete understanding of the roles of lncRNAs in cancer stemness of GC.

The present study discovered a novel lncRNA (LINC00853) that was obviously upregulated in GC tissues and cell lines. The dysregulated LINC00853 was associated with poor prognosis and TNM stage in GC patients. Functional experiments demonstrated that alteration of LINC00853 expression contributed to the GC cell viability, migration, sphere-forming ability and the expression of stemness markers (KLF4, NANOG, OCT4 and SOX2). Mechanistically, LINC00853 facilitated the transcription of PDZK1IP1 by directly binding to the transcription.factor FOXP3. Taken together, the results may suggest novel insights into the regulation of lncRNAs on cancer stemness and progression in GC. LINC00853 might serve as a new effective therapeutic target for GC treatment.

## Materials and methods

### Clinical samples

Tissue specimens of GC and normal samples were collected from patients in Hospital of Chengdu University of TCM (Chengdu, China). The clinical information of these patients is presented in Table 1. Throughout the experiment, informed consent was obtained from all patients. The Institutional Review Board of the Hospital of Chengdu University of TCM (Chengdu, China) (approval no. 2018KL-023) approved all experimental procedures, which were in compliance with the World Medical Association's Declaration of Helsinki.

**Table 1:** Clinical characteristics of GC patients

Feature	Normal	GC
Age		
< 50	13	11
≥ 50	12	14
Gender		
Male	12	15
Female	13	10
Tumor size (cm)		
< 5	/	9
≥ 5	/	16
Tumor stage		
I/II	/	17
III/IV	/	8
Lymphatic metastasis		
Yes	/	16
No	/	9
Distant metastasis		
Yes	/	15
No	/	10

### Cell culture and transfection

Four GC cell lines (SGC7901, AGS, MGC803, MKN-78) and human gastric epithelium GES-1 cell line were purchased from Cell Bank of the Chinese Academy of Sciences (Shanghai, China). AGS cells was cultured in F12K medium (Gibco, NY, USA) along with 10% fetal bovine serum (FBS, Invitrogen, MA, USA). Other cells were grown in RPMI-1640 medium (Invitrogen, MA, USA) with 10% FBS. Lentiviral plasmids overexpressing LINC00853/ PDZK1IP1/FOXP3 were constructed using pcDNA3.1 vector (GenePharma, Shanghai, China) following the producer’s instructions. The primers sequences used for plasmid construction were listed in Table S1. A co-transfection of lentiviral plasmids with packaging plasmids was used to produce lentiviral particles in 293 T cells. The shRNA lentivirus targeting LINC00853/ PDZK1IP1/FOXP3 were all constructed using pLKo.1 vector by Shanghai GenePharma Biotechnology company. An overview of all shRNA sequences had been provided in Table S2. The cells were harvested 48 h after cell transfection and subjected to efficiency determination via RT-PCR experiment.

### CCK-8

Using theCCK-8 assay (KeyGEN, Nanjing, China), cell proliferation was measured. Lentivirus infected MGC803 and MKN-78 cells were plated on 96-well plates (1 × 10^4^ cells/well) in triplicate and cultured for 48 h. Following putting in 10 µL CCK-8 reagent, further incubation for 1.5 h was completed. A microplate reader (BioRad 680, BioRad Laboratories, Hercules, CA, USA) was utilized to clarify the optical density under 450 nm.

### Transwell assay

Transwell assay was applied using 24-well Transwell chambers (Corning, NY, USA). Following transfection for 48 h, MGC803 and MKN-78 cells (1 × 10^5^ cells) were seeded on the upper chamber and 200 µL RPMI-1640 medium with 10% FBS were supplemented to the lower chamber to act as a chemoattractant. A paraformaldehyde (4%) solution was applied to fix the cells on the bottom chambers and subjected to stain using 1% crystal violet (Sigma, Shanghai, China) for 5 min at room temperature. Finally, each group was observed with three fields using an optical microscope (BX60, Olympus, Tokyo, Japan).

### Serum-free spheroid formation assay

Serum-free spheroid formation experiment was carried out in accordance with previous descriptions [17, 18]. Briefly, MGC803 and MKN-78 cells (1000 cells/well) were grown in 6 well ultra-low adherent culture plates (Corning-Co-Star) with serum-free culture medium containing B27 (2%, Sigma), EGF (20 ng/ml, Sigma) and FGF (10 ng/ml, Sigma). After 7 days, a light microscope (BX60, Olympus, Tokyo, Japan) was applied to photograph and count the cell spheres.

### Flow cytometry detection of CD44

CD44 positive cell proportions were investigated by flow cytometry using a FACSCalibur (BD Biosciences, California, USA). Briefly, after harvesting the cells with 0.25% trypsin–EDTA, they were washed and resuspended in PBS along with 0.5% FBS. Cell suspension and anti-CD44 antibody (dilution 1:200, cat. no. 550989, BD Biosciences, USA) were mixed and cultured for 1 h at room temperature. Flow cytometry was used to measure cells after collecting and suspending them in PBS.

### Reverse transcription-quantitative polymerase chain reaction (RT-PCR)

Following the producer’s protocol, RNA was separated via Trizol reagent (Invitrogen, MA, USA). After reverse transcription through PrimeScript RT Reagent Kit (Takara, Dalian, China) of RNA, complementary DNA (cDNA) was obtained. Next, PCR amplification was performed on 5 μL of cDNA using GAPDH as an internal parameter for LNC00853 and mRNA. The levels of genes were measured via applying the 2 − ΔΔCT method [19]. All primers applied for RT-PCR were designed and constructed via Sangon Biotech (Shanghai, China) and a list of the sequences was provided in Table S3.

### Western blot

Protein concentrations were quantified via a BCA protein concentration kit (Beyotime, Shanghai, China) after extracting the total protein with RIPA lysis buffer (Sigma, Shanghai, China). Using a 10% SDS-PAGE gels, we separated 25 μg of proteins and then transferred them to PVDF membranes (Merk, Darmstadt, Germany). Incubation with primary antibodies, including PDZK1IP1 (1:1500, ab156014, Abcam, Shanghai, China), FOXP3 (1:2000, ab4728, Abcam, Shanghai, China) and GAPDH (1:1000, AF7021, Affinity, Shanghai, China), was performed overnight at 4 °C after membranes had been blocked for 1 h using 5% fat-free milk. Following incubation with the horseradish peroxidase-labeled secondary antibody (1:1000, A0208, Beyotime, Shanghai, China) for 4 h at room temperature, the signal was detected via ECL reagents (Pierce, MA, USA) and subjected to image by the FluorChem imaging system (BioRad Laboratories, Hercules, CA, USA).

### Fluorescence in situ hybridization (FISH)

FISH test was applied to examine the localization of LINC00853 and FOXP3 in GC cell lines of MGC803 and MKN-78. Briefly, Specific probes to LINC00853 and FOXP3 were prepared and labeled with cy3 (GenePharma, China) and FITC (GenePharma, China), respectively. After mixing the probes with the pre-made hybridization buffer, samples were incubated overnight in this buffer. Finally, DAPI was applied to counterstain cell nuclei. The images were captured via a confocal laser-scanning microscopy (Zeiss, Jena, Germany).

### Immunohistochemistry (IHC)

As soon as the xenograft tumor tissues were collected, they were immersed in a paraformaldehyde solution for fixation. Then a paraffin embedding process followed after the tissues were dehydrated, cleared, and cleared. At 4 °C overnight, 4-μm paraffin sections were coated with specific antibody against Ki67 (1:200, AF0189, Affinity, Shanghai, China), followed by 1 h incubation with goat anti-mouse horseradish peroxidase (1:50, A0216, Beyotime, Shanghai, China). Subsequently, the slides were stained with DAB and counterstained with hematoxylin for 30 s, followed by conventional treatments. Finally, under the observation of a fluorescence microscope (BX60, Olympus, Tokyo, Japan), photographs were taken. By using ImageJ software, we calculated the positive staining area.

### RNA pull-down assay

LINC00853 and its antisense RNA were synthesized by Sangon (Shanghai, China) and biotin-labeled via the Biotin Labeling Kit (E-LK-B002, Elabscience, Texas, USA). Then a Pierce Magnetic RNA–Protein Pull-Down Kit (20,164, Thermo Scientific, USA) was applied to conduct RNA pull-down experiment. Briefly, biotin-labeled LINC00853, along with its anti-sense RNA, were incubated with cell lysates and streptavidin magnetic beads. Next, the precipitate was centrifuged, eluted via high-salt buffer, and then supernatant was gathered. Finally, the input and pulldown complex were subjected to western blot analysis.

### RNA immunoprecipitation (RIP) assay

RIP was applied via a Magna RIP RNA-Binding Protein Immunoprecipitation Kit (17–700, Millipore, Massachusetts, USA). Cells were lysed with of RIP lysate (P0013B, Beyotime, Shanghai, China) on ice for 30 min, then centrifuged at 14,000 rpm to collect supernatants. Then, the magnetic beads were thoroughly mixed with RIP Wash Buffer and subjected to incubate with rabbit anti-Ago2 antibody (ab186733, 1:50, Abcam, Shanghai, China) for 6 h at 4 °C. Re-suspended magnetic beads-antibody complex were incubated with 100 μL of cell supernatant overnight at 4 °C after washing in 900 μL of RIP Wash Buffer. Immunoprecipitated RNA was detected through RT-PCR analysis. Rabbit anti-IgG antibody (ab172730, 1:100, Abcam, Shanghai, China) was uses as a negative control (NC).

### Xenograft tumorigenesis

BALB/c nude mice (6–8 weeks old) were purchased from the Shanghai Laboratory Animal Center of China (Shanghai, China). The animals were randomly allocated into groups and kept on a normal diet with free access to water. A number of three mice were used per group. MGC803 and MKN-78 cells (4 × 10^5^) stably expressing sh-LINC00853 or LINC00853 OE were transplanted subcutaneously into the side of the back of each mouse. We monitored tumor growth weekly, and tumor size was calculated via applying the formula (length × width × width /2) [20]. Following the euthanasia of all the mice after four weeks, the tumors were collected and weighed. Three representative tumors were shown. All the animal experiments were approved by the Institutional Review Board of the Hospital of Chengdu University of TCM (Chengdu, China) (approval no. 2018KL-023).

### Statistical analysis

A statistical analysis of the data was carried out using GraphPad prism 7.0 version (GraphPad Software, CA, USA). All data are displayed as mean ± standard deviation (SD). An analysis of statistical significance was carried out through Student's t-test or one-way ANOVA. Correlations were quantified using Pearson correlation coefficients, and survival was evaluated using Kaplan–Meier analysis. Normal distribution was assumed for all data. It was set to 0.05 for statistical significance.

## Results

### LINC00853 exhibited high expression in GC and correlates with poor prognosis

**Fig. 1:**
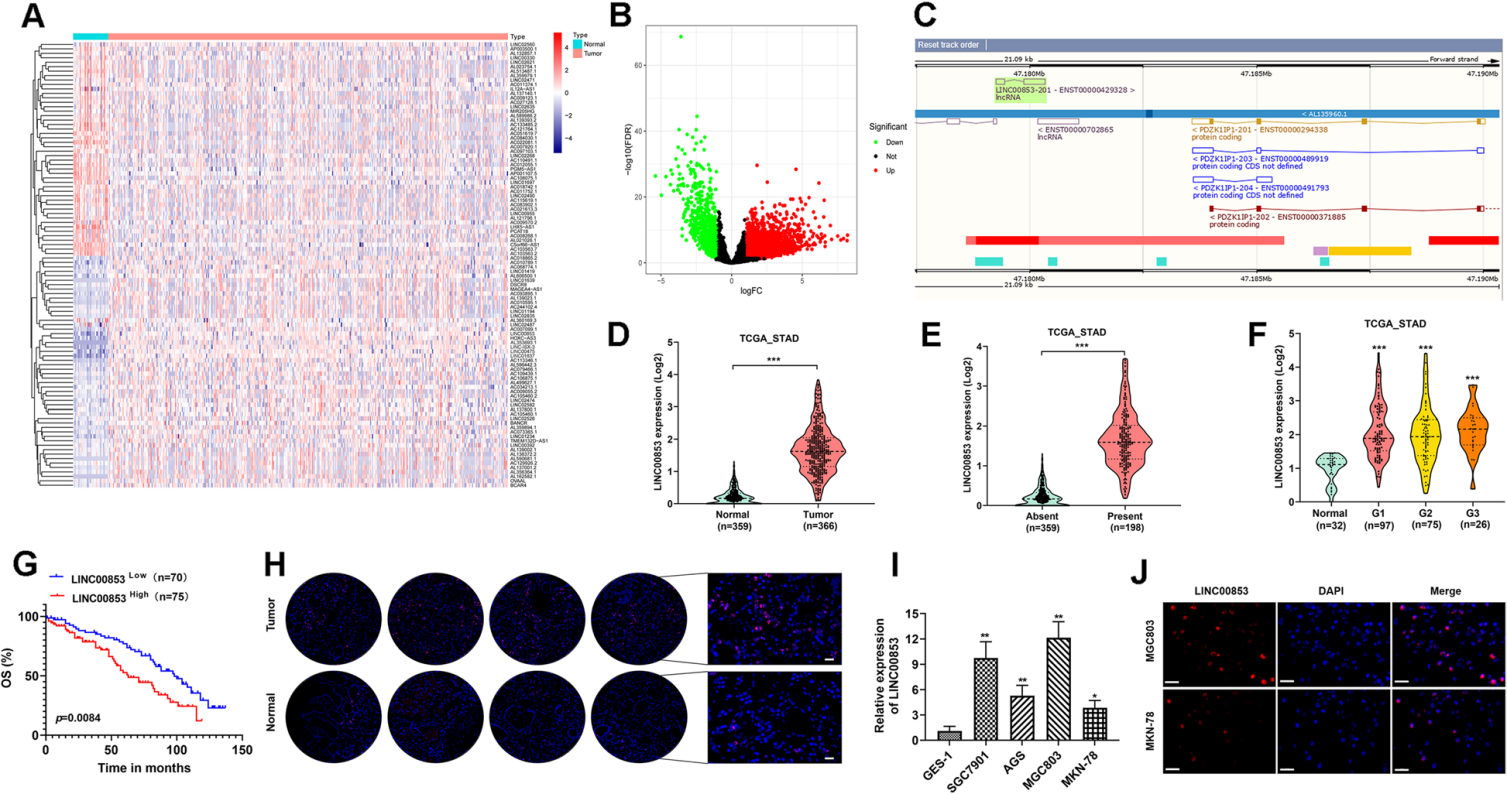
LINC00853 exhibited high expression in GC and was related to poor prognosis. **A** Heatmap of the top 100 upregulated or down-regulated differentially expressed lncRNAs from TCGA GC dataset. **B** The volcano plot of the lncRNAs in The TCGA dataset. **C** LINC00853 and PDZK1IP1 genomic loci from Ensembl database. **D** The level of LINC00853 in 366 GC tumor tissues and 359 normal tissues were obtained from TCGA database. **E** The correlation between LINC00853 expression and lymph node metastasis of GC patients. **F** The correlation between LINC00853 level and tumor stages of GC patients. **G** Kaplan–Meier overall survival curves in LINC00853 low and high expression of GC patients. **H** The level of LINC00853 in GC was examined by in situ hybridization on GC clinical samples. Scale bar = 50 μm. **I** The level of LINC00853 was clarified via RT-PCR analysis in 4 GC cell lines (SGC7901, AG5, MGC803, MKN-78). GES-1: normal gastric cell. **J** A FISH test was conducted to examine the cellular localization of LINC00853 in MGC803 and MKN-78 cells. DAPI was applied to localize cell nuclei. Scale bar = 50 μm. *p < 0.05, **p < 0.01, ***p < 0.001

To identify the dysregulated lncRNAs in GC, we analyzed the lncRNA expression profiles from The Cancer Genome Atlas (TCGA) database (https://tcga-data.nci.nih.gov/) and the results showed the top 100 down-regulated and up-regulated differentially expressed lncRNAs (Fig. 1A, B). Five top-five up-regulated lncRNAs were selected and subjected to RT-PCR validation in seven adjacent normal tissues, premalignant tissues and GC tissues. As result shown in Fig. S1, LINC00853 were mostly highly upregulated in GC tissues and selected for the following study. According to the information of Ensembl database (Fig. 1C), LINC00853 is located upstream of gene PDZK1IP1 (MAP17), and PDZK1IP1 is also found to be highly expressed in tumors and related to tumor cell stemness [21, 23]. We used GEPIA2 to analyze TCGA data and found LINC00853 showed an up-regulated level in GC samples compared with normal tissues (Fig. 1D). Besides, the over-expression of LINC00853 was strongly associated with lymph node metastasis (Fig. 1E). Furthermore, Fig. 1F demonstrated that the different expression of LINC00853 between clinical stages, with substantially higher level in advanced stage GC patients. Importantly, Kaplan–Meier curve revealed that GC patients with lower LINC00853 level had better overall survival than patients with higher LINC00853 levels (Fig. 1G). Similarly, the level of LINC00853 investigated via in situ hybridization on GC clinical samples was higher than that in normal tissues (Fig. 1H). Subsequently, we evaluated 4 GC cell lines (SGC7901, AG5, MGC803, MKN-78) by RT-PCR analysis to quantify the expression of LINC00853. As shown in Fig. 1I, MGC803 cells represented the highest LINC00853 expression, while the lowest was obtained in MKN-78 cells. In addition, FISH results unveiled that LINC00853 was primarily found in the nucleus of MGC803 and MKN-78 cells (Fig. 1J). Hence, there is a possibility that LINC00853 might play a critical role in GC promotion.

### LINC00853 motivated the proliferation, migration and stemness of GC cells

**Fig. 2:**
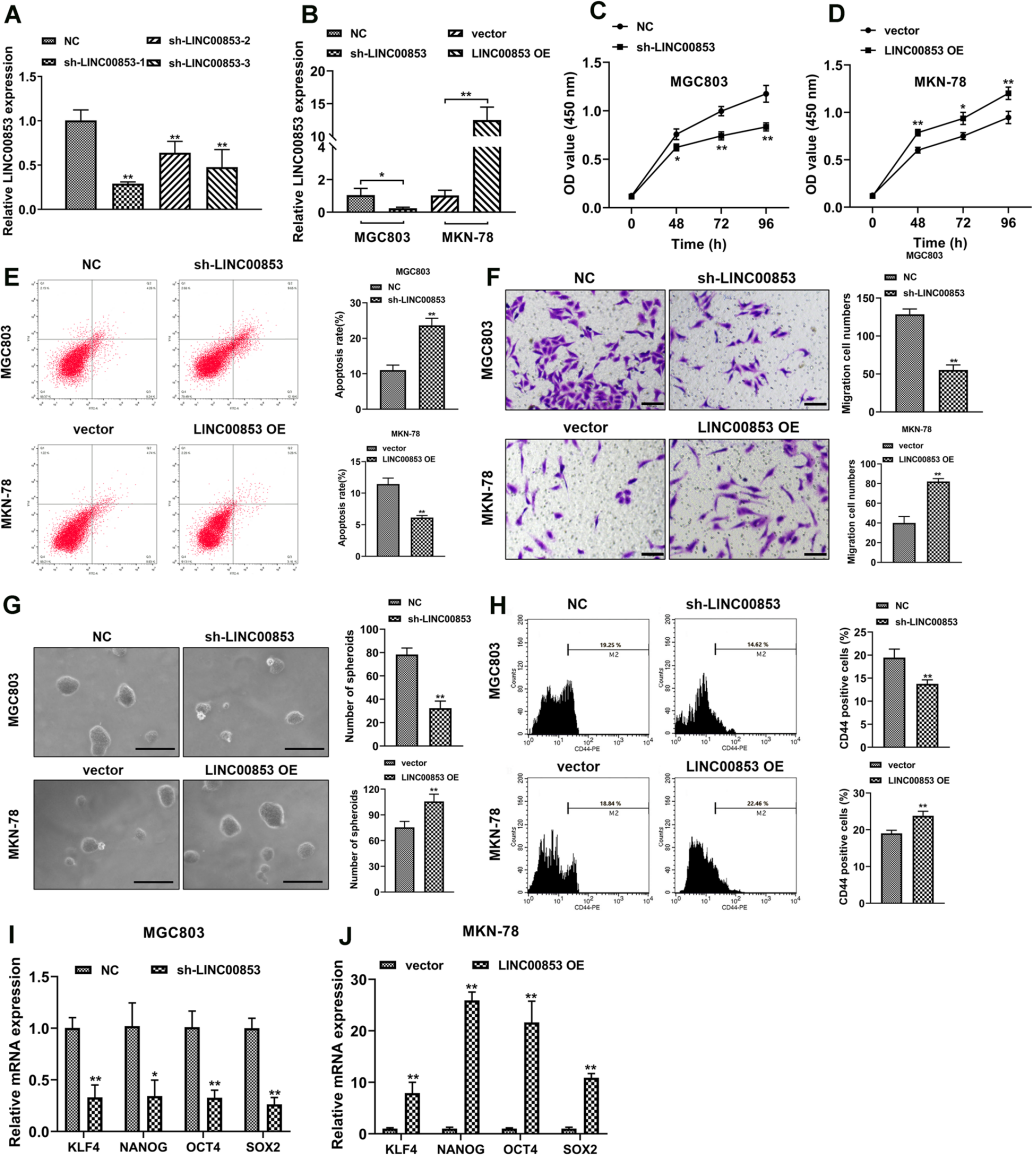
LINC00853 enhanced the proliferation, migration and stemness of GC cells. **A** MGC803 cells were transfected with lentiviruses encoding either the blank vector or LINC00853 shRNA (sh-LINC00853). The interfering efficiency of the lentivirus encoding sh-LINC00853 were confirmed by RT-PCR analysis. **B** The level of LINC00853 was clarified via RT-PCR after down/ up regulation of LINC00853 in MGC803 and MKN-78 cells, respectively. **C**, **D** CCK-8 test was applied to assess cell viability at 0, 48, 72, and 96 h time points. **E** The apoptosis of MGC803 and MKN-78 cells was investigated via staining with Annexin V/PI, followed by flow cytometry analysis. **F** Migration ability of cells examined via Transwell assay. Scale bar = 100 μm. **G** The effect of knockdown or overexpression of LINC00853 on serum-free sphere formation was evaluated using a tumor sphere formation assay. Scale bar = 100 μm. **H** The CD44 positive proportion was determined by flow cytometry. **I**, **J** The levels of stemness-related factors KLF4, SOX2, NANOG, and OCT4 were detected through RT-PCR analysis. *p < 0.05, **p < 0.01

To deeply explore the biological influences of LINC00853 in vitro, we used shRNA-packed lentivirus to knockdown the expression of LINC00853. RT-PCR test was applied to quantify the transfection efficiency and sh-LINC00853-1 was chosen for following tests due to its highest interference efficiency (Fig. 2A). The lentivirus conveying blank vector and LINC00853-overexpressing vector were constructed and the overexpression efficiency was examined via RT-PCR after transfection into MKN-78 cells (Fig. 2B). Next, MGC803 and MKN-78 cells were transfected with sh-LINC00853 and LINC00853 OE, respectively, and CCK-8 test was carried out to investigate cell viability at indicated time points (0, 48, 72 and 96 h). The results unveiled that the viability of MGC803 cells transfected with sh-LINC00853 decreased, while the viability of MKN-78 cells with LINC00853 overexpression increased (Fig. 2C, D). Moreover, flow cytometry analysis clarified that LINC00863 suppression remarkably induced cell apoptosis, which was attenuated by LINC00853 overexpression (Fig. 2E). Besides, cell migration ability was assessed and as Fig. 2F shown, down-regulation of LINC00853 obviously suppressed the migration ability of MGC803 cells. Whereas, up-regulation of LINC00853 effectively promoted the migration of MKN-78 cells. Furthermore, the serum-free sphere formation assay revealed that LINC00853 knockdown reduced tumor spheres growth while overexpression of LINC00853 facilitated spheres growth (Fig. 2G). Flow cytometry analysis revealed the inhibited CD44 positive proportions with sh-LINC00853 transfection. In contrast, overexpression of LINC00853 increased the proportion of CD44 positive cells (Fig. 2H). The levels of stemness-related factors KLF4, SOX2, NANOG, and OCT4 was reduced in LINC00853 knockdown MGC803 cells, while was promoted in LINC00853 overexpression MKN-78 cells **(**Fig. 2I-J). These findings suggested that LINC00853 was related to the cell migration and stem-like properties.

### Alteration of PDZK1IP1 expression reversed LINC00853-mediated progression and stemness in GC cells

**Fig. 3:**
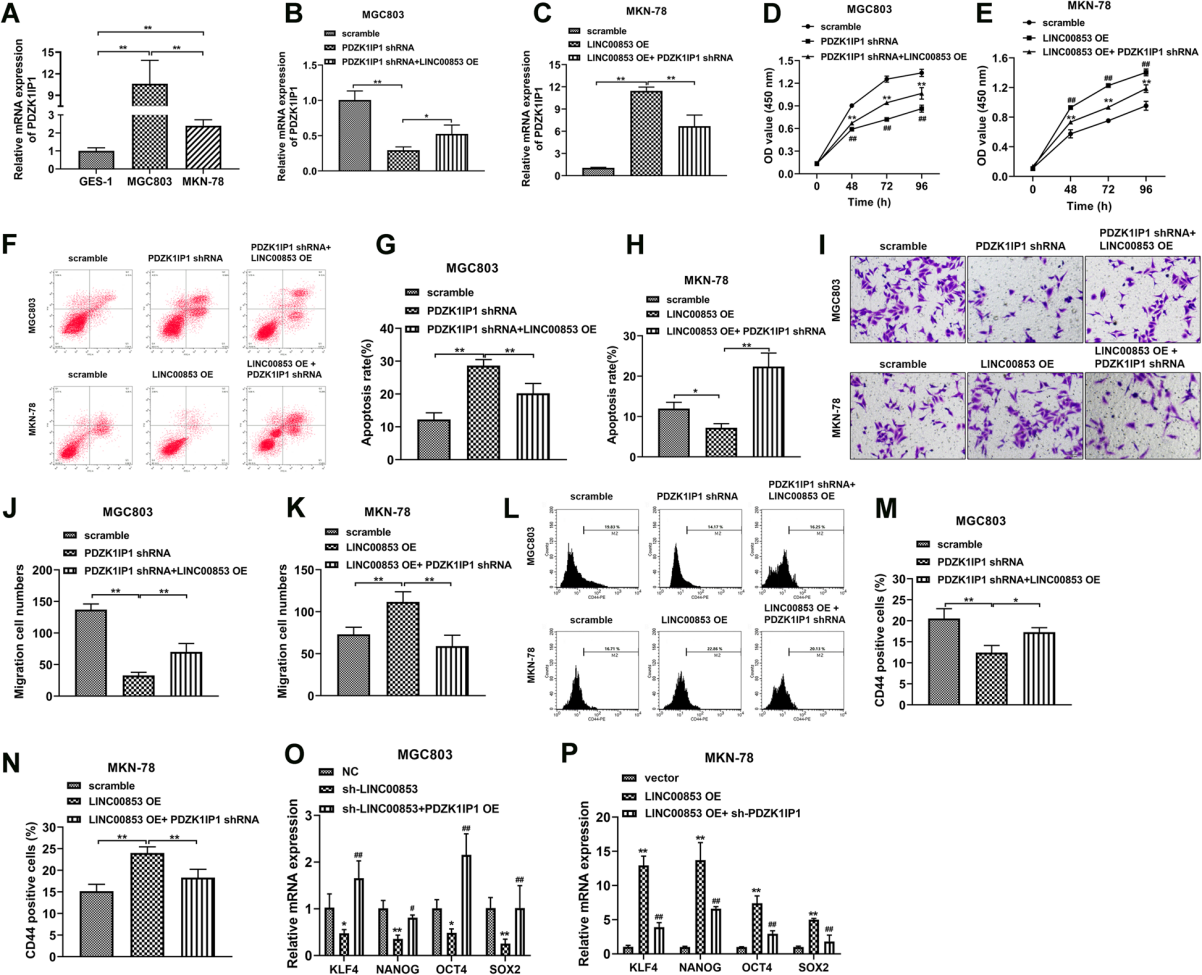
Alteration of PDZK1IP1 expression reversed LINC00853-mediated progression and stemness in GC cells. **A** RT-PCR was applied to investigate the level of PDZK1IP1 in MGC803 and MKN-78 cells. **B**, **C** The level of PDZK1IP1 was validated via RT-PCR experiment in MGC803 and MKN-78 cells after indicated transfection. **D**, **E** Proliferation curves of MGC803 cells transfected with sh-LINC00853 or/and PDZK1IP1 OE and MKN-78 cells with overexpression of LINC00853 or/and sh-PDZK1IP1, as determined by CCK8 assay. **F–H** Apoptosis assay of transfected MGC803 and MKN-78 cells by flow cytometry analysis of Annexin V-FITC/PI staining. **I-K** Transwell migration assay of MGC803 and MKN-78 cells following indicated transfection. Scale bar = 100 μm. **L-N** Flow cytometry assessment of CD44 positive proportion after transfection. **O**, **P** RT-PCR analysis of pluripotency genes, OCT4, NANOG, KLF4 and SOX2 in MGC803 and MKN-78 cells. *p < 0.05, **p < 0.01

LINC00853 is located upstream of the PDZK1IP1 gene locus in chromosome 1p33. Previous reports had unveiled that PDZK1IP1 is overexpressed in multiple of cancers and involved with tumor stemness [21–23]. Here we hypothesized that LINC00853 modulated tumor progression and stemness through regulation of PDZK1IP1. Firstly, RT-PCR analyzed the level of PDZK1IP1 in GC cells and the increased PDZK1IP1 level was observed in MGC803 and MKN-78 cells (Fig. 3A). Next, we designed and constructed the PDZK1IP1 shRNA and overexpression vector. The transfection efficiency was determined by RT-PCR analysis (Fig. S2). RT-PCR analysis represented higher level of PDZK1IP1 in LINC00853 OE MGC803 cells transfected with PDZK1IP1-expressing lentiviral construct (rescue) (Fig. 3B). Conversely, transfection with sh-PDZK1IP1 suppressed the expression of PDZK1IP1 induced by LINC00853 OE (Fig. 3C). Moreover, the cell viability, apoptosis, and cell migration ability were quantified through CCK-8, flow cytometry and Transwell assay, respectively. The findings revealed that the reintroduction of PDZK1IP1 remarkably reversed the cell viability (Fig. 3D, E), apoptosis (Fig. 3F-H) and migration ability (Fig. 3I-K) driven by LINC00853. Further studies demonstrated that overexpression of LINC00853 accelerated the CD44 positive cells proportion (Fig. 3L, M) and decreased the levels of stemness cell markers including KLF4, NANOG, OCT4 and SOX2 that were inhibited by sh-LINC00853 in MGC803 cells (Fig. 3O). In contrast, knockdown of PDZK1IP1 brought with the opposite results (Fig. 3L, N, P).

### LINC00853 promoted PDZK1IP1 expression through interacting with transcriptional factor FOXP3

**Fig. 4:**
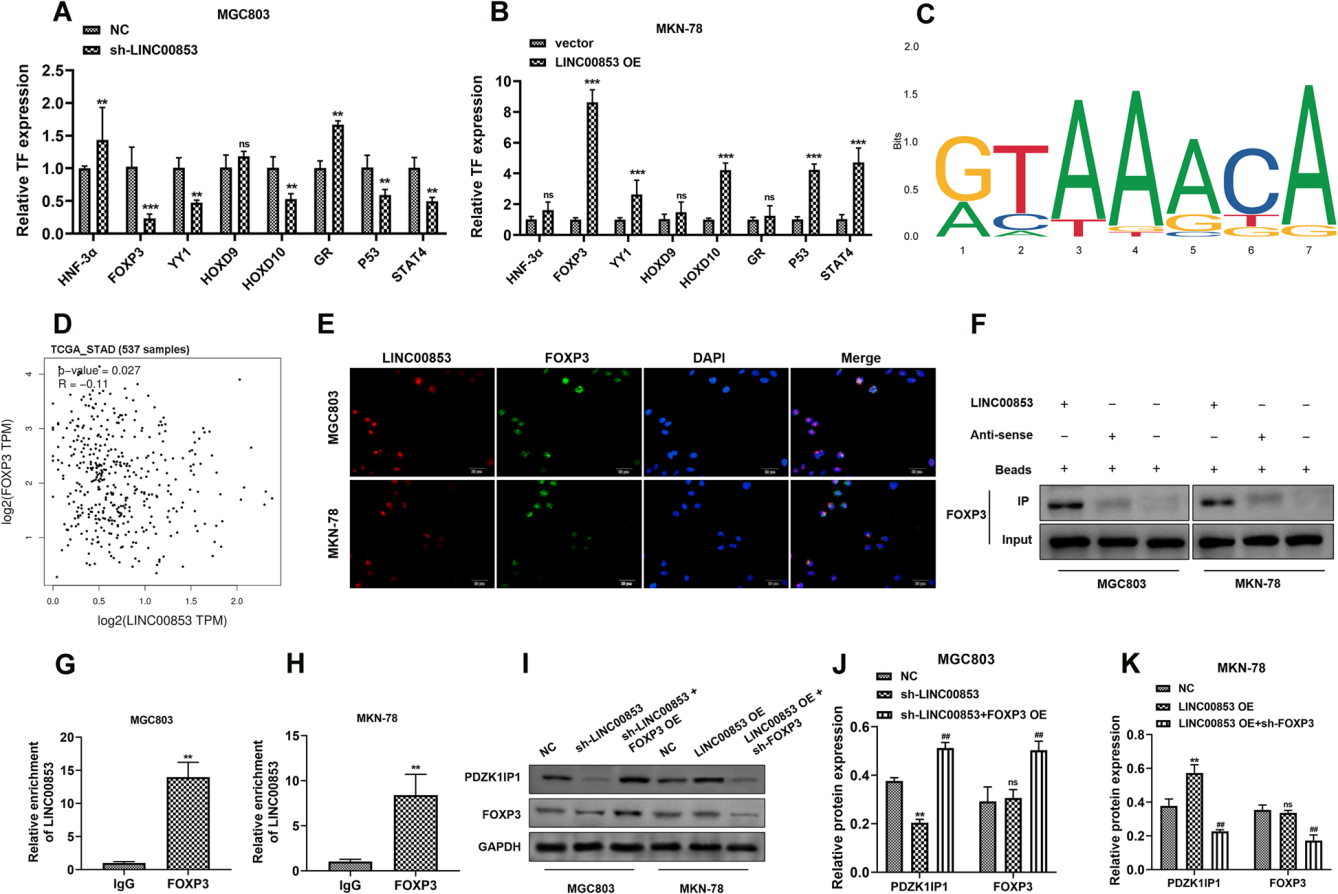
LINC00853 promoted PDZK1IP1 expression by interacting with transcriptional factor FOXP3. **A**, **B** The expression levels of the potential transcription factors predicted by UCSC and PROMO database were validated by RT-PCR after transfection with sh-LINC00853 or LINC00853 OE, and FOXP3 gene expression was observed as most down/up-regulated. **C** DNA motif of FOXP3 predicted from JASPAR. **D** The correlation between LINC00853 expression and FOXP3 expression in GC patients, as analyzed in GEPIA database. **E** The colocalization of LINC00853 and FOXP3 in GC cells was examined via FISH assay. Scale bar = 20 μm. RNA pulldown (**F**) and RIP assay (**G-H**) results demonstrating the interaction between LINC00853 and FOXP3. **I-K** Western Blots assay was carried out to determine the expression of PDZK1IP1 and FOXP3 after indicated transfection. *p < 0.05, **p < 0.01, ns = non-significant

To further illustrate the transcriptional regulation of LINC00853 in GC, we predicted potential transcription factors that might interact with LINC00853 promoter region using UCSC and Promo database (Table S4), and eight candidate transcription factors were screened by RT-PCR assay. As shown in Fig. 4A, FOXP3, YY1, HOXD10, P53 and STAT4 were down-regulated after sh-LINC00853 transfection, with the maximal reduction of FOXP3. In MKN-78 cells, the most FOXP3 mRNA level elevation was observed on transfection with LINC00853 OE (Fig. 4B). Based on the JASPAR website, the DNA motif of FOXP3 and possible binding sites of FOXP3 in the LINC00853 promoter region were predicted (Fig. 4C). Next, the positive correlation of LINC00853 and FOXP3 expression was analyzed by GEPIA database (Fig. 4D). Additionally, FISH experiment unveiled the co-localization in the nucleus of LINC00853 and FOXP3 in GC cells (Fig. 4E). To confirm the interaction between LINC00853 and FOXP3, RNA pulldown was conducted and the results determined that biotin-labeled LINC00853 enriched FOXP3 (Fig. 4F). Moreover, the interaction between LINC00853 and FOXP3 was further confirmed by RIP assay (Fig. 4G-H). We designed and constructed the FOXP3 shRNA and overexpression vector. The transfection efficiency was evaluated by RT-PCR analysis (Fig. S3). Western blot assay (Fig. 4I-K) represented that knockdown of LINC00853 in MGC803 cells obviously inhibited the level of PDZK1IP1, while no significant difference in FOXP3 expression was observed. And co-transfection of sh-LINC00853 and FOXP3 OE promoted the levels of PDZK1IP1 and FOXP3. Reciprocally, inhibition of FOXP3 expression obtained the opposite results. Taken together, these data indicated that LINC00853 specifically bound to transcription factor FOXP3, thereby regulating transcription of PDZK1IP1.

### FOXP3-mediated regulation increased GC progression and stemness

**Fig. 5:**
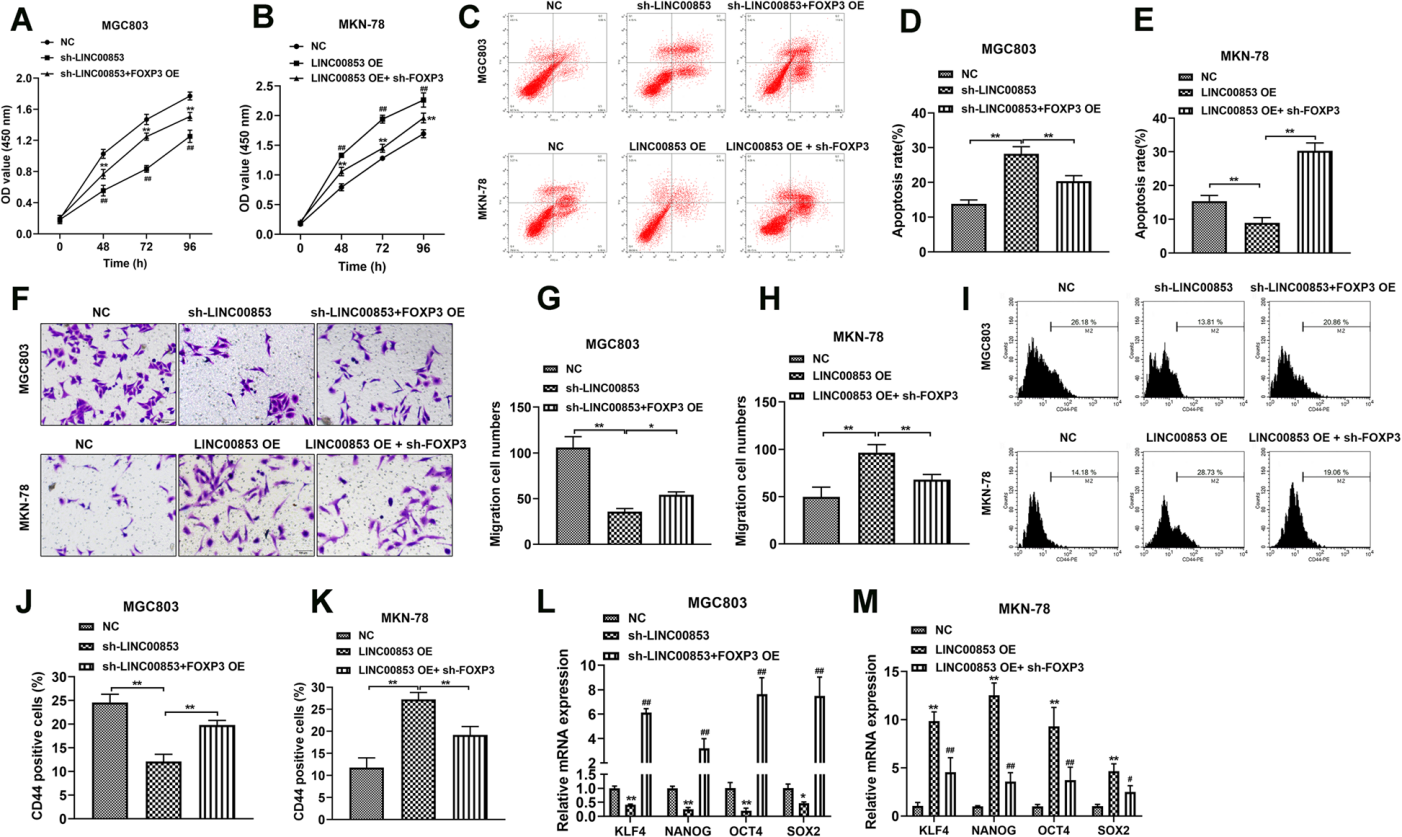
FOXP3-mediated regulation promoted GC progression and stemness. **A**, **B** Proliferation curves of MGC803 cells transfected with sh-LINC00853 or/and FOXP3 OE and MKN-78 cells with overexpression of LINC00853 or/and sh-FOXP3, as determined by CCK8 assay. **C-E** Flow cytometry quantitation of transfected MGC803 cells and MKN-78 cells after indicated transfection. F–H Transwell assay was conducted to evaluate the cell migration of transfected MCG803 and MKN-78 cells. Scale bar = 100 μm. I-K Flow cytometry assessment of CD44 positive proportion of transfected cells. (L-M) RT-PCR analysis to identify the expression levels of stemness genes, including OCT4, NANOG, KLF4 and SOX2. *p < 0.05, **p < 0.01

To explore the specific mechanism by which LINC00853 and FOXP3 influence the biological properties of GC cells, MFC803 cells were transfected with sh-LINC00853 and sh-LINC00853 + FOXP3 OE, respectively. MKN-78 cells were transfected with LINC00853 OE and LINC00853 OE + sh-FOXP3, respectively. CCK-8, flow cytometry and Transwell assay were utilized to quantify the change of cell viability, apoptosis and migration ability. The results indicated that overexpression of FOXP3 in MGC803 cells significantly reversed the sh-LINC00853-modulated cell viability (Fig. 5A), apoptosis (Fig. 5C, E) and migration (Fig. 5F, G). In MKN-78 cells, knockdown of FOXP3 remarkably reversed LINC00853 OE-induced biological properties (Fig. 5B, D, E-F, H). Moreover, up-regulation of FOXP3 increased the CD44 positive cells proportion (Fig. 5I-J) and attenuated the expression of stemness cell markers of KLF4, NANOG, OCT4 and SOX2 which were suppressed by sh-LINC00853 in MGC803 cells (Fig. 5L). In contrast, down-regulation of FOXP3 achieved the opposite results (Fig. 5I, K, M).

### LINC00853 significantly accelerated tumor progression of GC cells in vivo

**Fig. 6:**
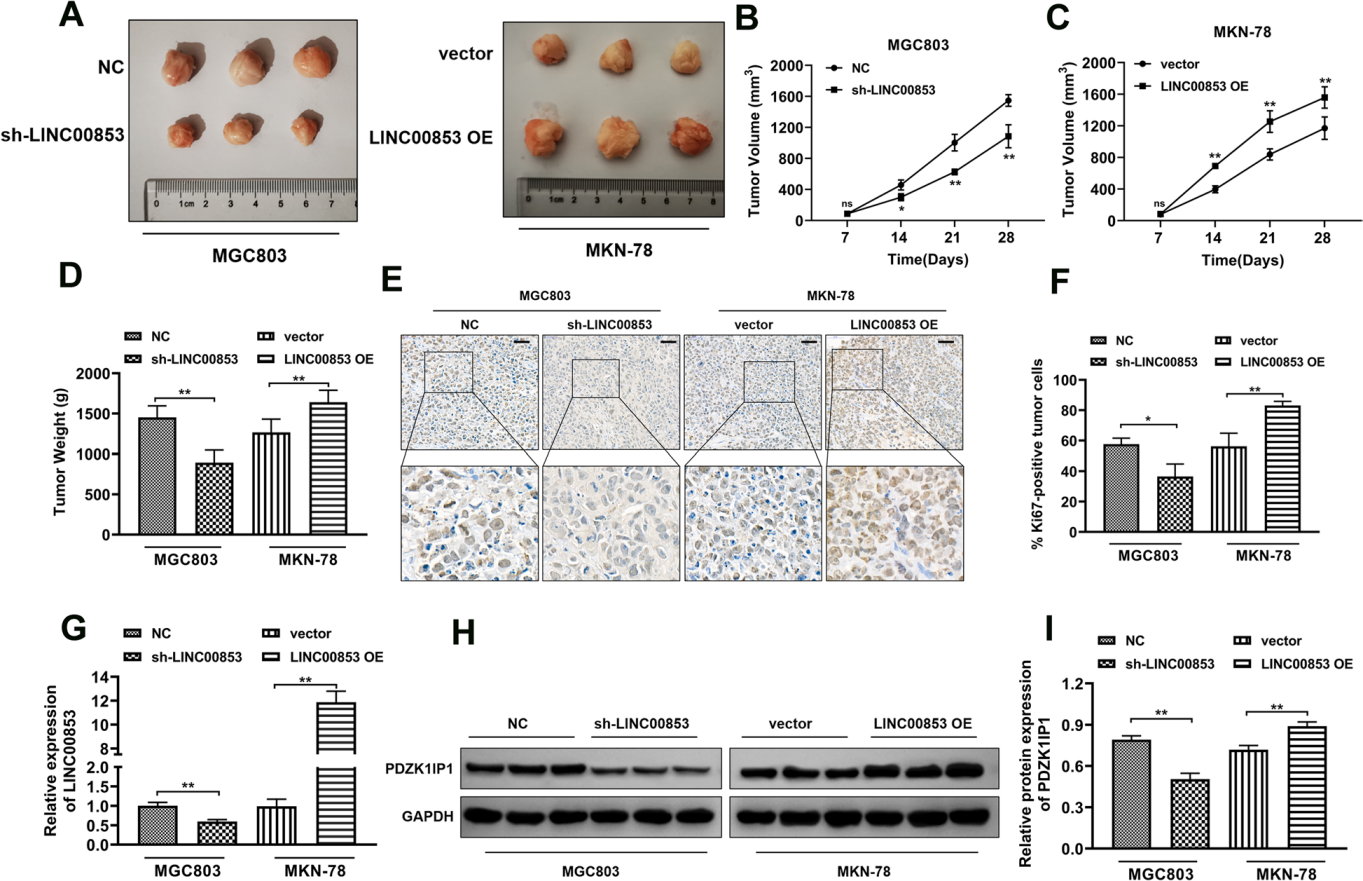
LINC00853 significantly accelerated tumor progression of GC cells in vivo. The photos of the tumor xenografts (**A**) and the tumor volume curve (**B-C**) and the tumor weight graph (**D**) of xenograft tumors generated via subcutaneous administration of MGC803 and MKN-78 cells stably transfected with sh-LINC00853 or LINC00853 OE, respectively. **E** Detection of proliferation in tumor tissues with Ki67 staining assay. Scale bar = 50 μm. **F** ImageJ software was utilized to quantify the number of Ki67-positive cells in tumor tissue. **G** The level of LINC00853 was clarified via RT-PCR test. **H**, **I** Western blot assay was applied to illustrate the level of PDZK1IP1. *p < 0.05, **p < 0.01

Next, we evaluated the regulation of LINC00853 in tumor growth in vivo. Stable cell strains of MGC803 with knockdown of LINC00853 and MKN-78 with overexpression of LINC00853, and then subcutaneous tumor implantation was performed using the cell strains. Results for tumor morphology (Fig. 6A), growth curves (Fig. 6B, C), and weight (Fig. 6D) revealed that down-regulation of LINC0085 effectively inhibited tumor growth in mice, while up-regulation of LINC00853 remarkably promoted tumor progression. Besides, immunohistochemical analysis for Ki67 indicated that knockdown of LINC00853 dramatically facilitated Ki67 levels, while overexpression of LINC00853 notably inhibited Ki67 expression in tumor tissues (Fig. 6E, F). Moreover, RT-PCR and Western blot assay were conducted to confirm the level of LINC00853 and PDZK1IP1. The findings displayed the decreased LINC00853 in sh-LINC00853 group while the increased expression in LINC00853 OE group (Fig. 6G). The expression of PDZK1IP1 (Fig. 6H, I) were consistent with the trend of LINC00853. These findings unveiled that the inhibitor of LINC00853 might be a promising effective therapeutic target for treatment on GC patients.

## Discussion

LncRNAs have been identified as crucial regulators in multiple cancers and contribute to tumor initiation and development [24–26]. But the regulatory network and specific mechanisms of lncRNAs in GC have not yet been fully elucidated. LNC00853 is a lncRNA located on chromosome 1p33 with an 1826 nucleotide length. LINC00853 has been reported to regulate the level of CC chemokine receptor 9 (CCR9) modulating the binding of CCR9 and CC chemokine ligand 25 (CCL25), which was involved in the process of the infiltration of T cell acute lymphoblastic leukemia [27]. In the early stages of hepatocellular carcinoma, serum small extracellular vesicle derived LINC00853 may provide a new diagnostic biomarker [28]. However, the biological roles of LINC00853 in GC are not explored. In this study, we identified that LINC00853 as an up-regulated lncRNA in GC, acted crucial roles in disease development and unsatisfied prognosis. In vivo and in vitro functional experiments, we revealed that LINC00853 promotes GC proliferation, migration, and tumor stemness. Furthermore, LINC00853 could activate the transcription of PDZK1IP1 by interacting with the transcriptional factor FOXP3.

The PDZK1IP1 (MAP17) protein is a small (~ 17 kDa), non-glycosylated protein associated with cell-to-cell interactions [29, 30]. In PDZK1IP1, the C-terminal PDZ domain enables STPM to function as a transport vehicle for amino acids from the Golgi to the cell membrane [31]. It has been shown to be a gene that is commonly upregulated in tumors, being overexpressed in more than 50% of tumors analyzed in advanced tumors or metastases [32]. MAP17 upregulation activated Notch pathway through segregating NUMB, which caused direct alteration of tumor sphere formation and Notch and Stem pathway transcription [21]. It has been found that MAP17 levels estimate sensitivity to standard treatments, such as platinum compounds, EGFR inhibitors and proteasome inhibitors [33]. However, the influences and specific mechanism of PDZK1IP1 in GC have been rarely discovered.

To summarize, this study determined that LINC00853 was up-regulated in GC in vivo and in vitro. Mechanistically, LINC00853 promoted PDZK1IP1 expression through binding to FOXP3, thus maintaining tumor stemness and accelerating tumor progression. Therefore, our findings might suggest novel GC prevention and therapeutic strategies in the future. Further explorations need to be carried out to validate our findings and identify potential clinical utility.

## Conclusions

LINC00853 exerted a cancer-promoting role in GC through FOXP3-mediated transcription of PDZK1IP1. Hence, LINC00853 may provide a promising novel strategy for GC patients.

## Supplementary Information

**Additional file 1:**  **Table S1. **Primers sequencesused for plasmid construction. **Table S2. s**hRNAsequences used in this study. **Table S3. **Primersused for RT- PCR in this study. **Table S4.** List of LINC00853 target transcription factors byPROMO database. **Fig S1.** RT-PCRvalidation of AL353693.1, LINC-ISX-3, LINC00475, LINC00853, and LINC01637 in 7adjacent normal tissues, premalignant tissues and GC tissues. N: adjacentnormal tissues; P: Premalignant tissues; T: GC tissues. The tissue sampleswere collected from patients in Hospital of Chengdu University of TCM (Chengdu,China). *p<0.05; **p<0.01. **Fig S2.** The transfectionefficiency of PDZK1IP1 shRNA and PDZK1IP1overexpression lentiviruses was validated by RT-PCR in MGC803 (A) and MKN-78 (B)cells. (n=3). Data were presented as mean ± SD. **p<0.01. **Fig S3.** The transfectionefficiency of FOXP3 shRNA and FOXP3 overexpression lentiviruses was validatedby RT-PCR in MGC803 (A) and MKN-78 (B) cells. (n=3). Data werepresented as mean ± SD. **p<0.01.

## Data Availability

The datasets used and/or analyzed during the current study are available from the corresponding author on reasonable request.

## Abbreviations

GC: Gastric cancer
LncRNAs: Long non-coding RNAs
TCGA: The Cancer Genome Atlas
RIP: RNA immunoprecipitation
FOXP3: Forkhead Box P3
PDZK1IP1: PDZK1 interacting protein 1
CSCs: Cancer stem-like cells
IGF2BPs: Insulin-like growth factor 2 mRNA binding proteins
EMT: Epithelial-mesenchymal transition
CCR9: CC chemokine receptor 9
CCL25: CC chemokine ligand 25
