# Supplementary material for: LINC00853 contributes to tumor stemness of gastric cancer through FOXP3-mediated transcription of PDZK1IP1
Source: Biol Proced Online. 2023 Jul 4;25:20. doi: 10.1186/s12575-023-00213-2 (PMC10318836; doi:10.1186/s12575-023-00213-2)
Supplement: Supplementary file 1 — Additional file 1: Table S1. Primers sequencesused for plasmid construction. Table S2. shRNAsequences used in this study. Table S3. Primersused for RT- PCR in this study. Table S4. List of LINC00853 target transcription factors byPROMO database. Fig S1. RT-PCRvalidation of AL353693.1, LINC-ISX-3, LINC00475, LINC00853, and LINC01637 in 7adjacent normal tissues, premalignant tissues and GC tissues. N: adjacentnormal tissues; P: Premalignant tissues; T: GC tissues. The tissue sampleswere collected from patients in Hospital of Chengdu University of TCM (Chengdu,China). *p<0.05; **p<0.01. Fig S2. The transfectionefficiency of PDZK1IP1 shRNA and PDZK1IP1overexpression lentiviruses was validated by RT-PCR in MGC803 (A) and MKN-78 (B)cells. (n=3). Data were presented as mean ± SD. **p<0.01. Fig S3. The transfectionefficiency of FOXP3 shRNA and FOXP3 overexpression lentiviruses was validatedby RT-PCR in MGC803 (A) and MKN-78 (B) cells. (n=3). Data werepresented as mean ± SD. **p<0.01. [file 12575_2023_213_MOESM1_ESM.docx]

**Table S1** Primers sequences used for plasmid construction.

| **Primer sequence** |  |  |
| --- | --- | --- |
| LINC00853 | Forward | 5ʹ- ATAGGATCC GCCGCGCCTGAAGCTCAACTTCCGA -3ʹ |
|  | Reverse | 5ʹ- ATACTCGAG TGACACATGCAGAAATACTATCTGT -3ʹ |
| PDZK1IP1 | Forward | 5ʹ- GAGGGATCC ATGCCCAACCCCAGGCCTGGCAAGC -3ʹ |
|  | Reverse | 5ʹ- GAGCTCGAG TTACATCGGGGTGCTGCGGACCTTG -3ʹ |
| FOXP3 | Forward | 5ʹ- ATAGGATCC ATGCCCAACCCCAGGCCTGGCAAGC-3ʹ |
|  | Reverse | 5ʹ- ATA CTCGAG TCAGGGGCCAGGTGTAGGGTTGGAA -3ʹ |

**Table S2 s**hRNA sequences used in this study.

| **shRNAs** |  |
| --- | --- |
| sh-LINC00853-1 | 5′- GAAGCACAATAAACAGATA -3′ |
| sh-LINC00853-2 | 5′- GCATGATTCCCAAGCTCAA -3′ |
| sh-LINC00853-3 | 5′- CAGCAATAGTCCTGATCTA-3′ |
| sh-PDZK1IP1-1 | 5′- GGATGGATGTCCAGTGCTA-3′ |
| sh-PDZK1IP1-2 | 5′- CCTAATTTCTGTGAAATAA -3′ |
| sh-PDZK1IP1-3 | 5′- GGCCCTAATTTCTGTGAAA-3′ |
| sh-FOXP3-1 | 5′- GGACACTCAATGAGATCTA -3′ |
| sh-FOXP3-2 | 5′- GCTGGAGTTCCGCAAGAAA -3′ |
| sh-FOXP3-3 | 5′- CAGAGGAACTACTCATTTA-3′ |
| NC | 5′- GCTTCGCGCCGTAGTCTTA -3′ |

**Table S3** Primers used for RT- PCR in this study.

| **Primer sequence** |  |  |
| --- | --- | --- |
| LINC00853 | Forward | 5ʹ- CAGAAAAGCTCCCGAAACTG -3ʹ |
|  | Reverse | 5ʹ- AAGCTTGAGCTTGGGAATCA -3ʹ |
| KLF4 | Forward | 5ʹ- CCCACACAGGTGAGAAACCT -3ʹ |
|  | Reverse | 5ʹ- ATGTGTAAGGCGAGGTGGTC -3ʹ |
| NANOG | Forward | 5ʹ- TTCCTTCCTCCATGGATCTG -3ʹ |
|  | Reverse | 5ʹ- TCTGCTGGAGGCTGAGGTAT -3ʹ |
| OCT4 | Forward | 5ʹ- GTACTCCTCGGTCCCTTTCC -3ʹ |
|  | Reverse | 5ʹ- CAAAAACCCTGGCACAAACT -3ʹ |
| SOX2 | Forward | 5ʹ- ACACCAATCCCATCCACACT -3ʹ |
|  | Reverse | 5ʹ- GCAAACTTCCTGCAAAGCTC -3ʹ |
| PDZK1IP1 | Forward | 5ʹ- AGTCCTGGTGGGAACAGATG -3ʹ |
|  | Reverse | 5ʹ- ACTGGACATCCATCCCATGT -3ʹ |
| FOXP3 | Forward | 5ʹ-CCTCCCCCATCATATCCTTT -3ʹ |
|  | Reverse | 5ʹ-TTGGGGTTTGTGTTGAGTGA -3ʹ |
| GAPDH | Forward | 5ʹ-CGACCACTTTGTCAAGCTCA -3ʹ |
|  | Reverse | 5ʹ-AGGGGTCTACATGGCAACTG -3ʹ |

**Table.S4** List of LINC00853 target transcription factors by PROMO database

| Gene name | Target protein |
| --- | --- |
| LINC00853 | FOXP3 |
| LINC00853 | HNF-3α |
| LINC00853 | GR |
| LINC00853 | YY1 |
| LINC00853 | HOXD9 |
| LINC00853 | HOXD10 |
| LINC00853 | P53 |
| LINC00853 | STAT4 |


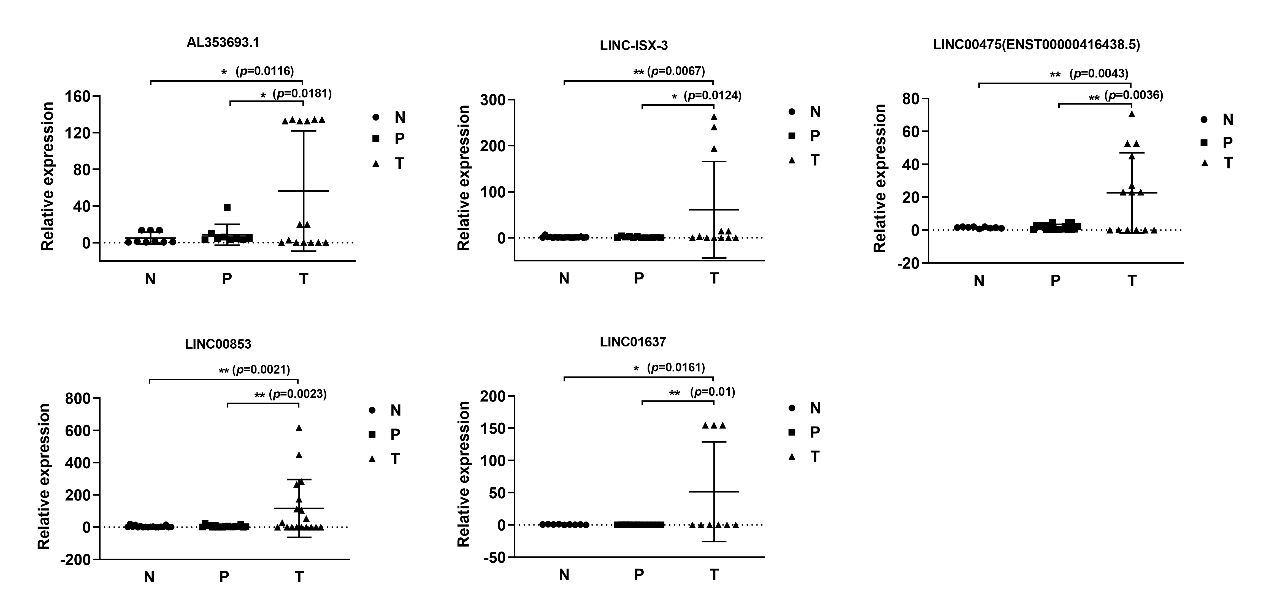


**Fig S1**. RT-PCR validation of AL353693.1, LINC-ISX-3, LINC00475, LINC00853, and LINC01637 in 7 adjacent normal tissues, premalignant tissues and GC tissues. N: adjacent normal tissues; P: Premalignant tissues; T: GC tissues. The tissue samples were collected from patients in Hospital of Chengdu University of TCM (Chengdu, China). **p*<0.05; ***p*<0.01.


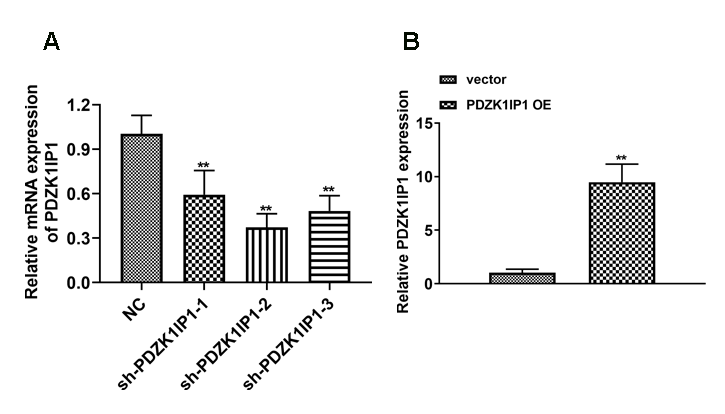


**Fig S2**. The transfection efficiency of PDZK1IP1 shRNA and PDZK1IP1 overexpression lentiviruses was validated by RT-PCR in MGC803 (A) and MKN-78 (B) cells. (n=3). Data were presented as mean ± SD. ***p*<0.01.


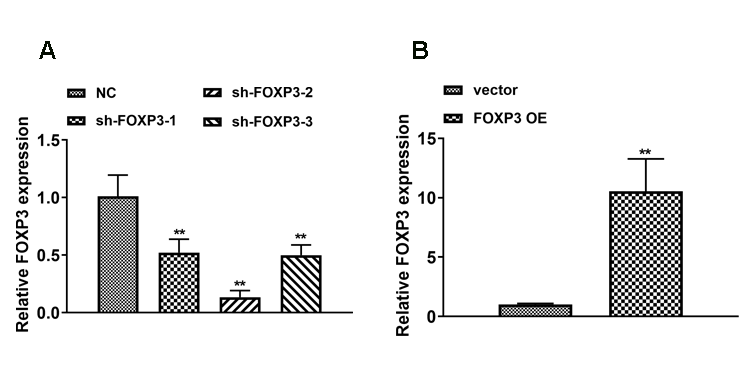


**Fig S3**. The transfection efficiency of FOXP3 shRNA and FOXP3 overexpression lentiviruses was validated by RT-PCR in MGC803 (A) and MKN-78 (B) cells. (n=3). Data were presented as mean ± SD. ***p*<0.01.
